# Microbiome transfer from native to invasive species may increase invasion risk

**DOI:** 10.1098/rspb.2024.1318

**Published:** 2024-11-06

**Authors:** Maria M. Martignoni, Oren Kolodny

**Affiliations:** ^1^ Department of Ecology, Evolution and Behavior, A. Silberman Institute of Life Sciences, Faculty of Sciences, Hebrew University of Jerusalem, Jerusalem 9190401, Israel

**Keywords:** host–microbe interactions, rapid adaptation, biological invasion, mathematical model, differential equations, microbiome

## Abstract

In a fast-changing world, understanding how organisms adapt to their environment is a pressing necessity. Research has focused on genetic adaptation, while our understanding of non-genetic modes is still in its infancy. The host-associated microbiome can be considered a non-genetic mode of adaptation, which can strongly influence an organism’s ability to cope with its environment. However, the role of the microbiome in host ecological dynamics is largely unexplored, particularly in animal communities. Here, we discuss the following hypothesis: invasive species may rapidly adapt to local conditions by adopting beneficial microbes from similar co-occurring native species. This occurs when the invader’s fitness is influenced by adaptation to local conditions that is facilitated by microbes acquired from native microbiomes. We present a minimal mathematical model to explore this hypothesis and show that a delayed acquisition of native microbes may explain the occurrence of an invasion lag. Overall, our results contribute to broadening the conceptualization of rapid adaptation via microbiome transfer and offer insights towards designing early intervention strategies for invasive species management.

## 1. Introduction

Invasive species cause annual damages of billions of dollars [1–3], and understanding the factors facilitating their adaptation is paramount for mitigating their impact. Early detection and eradication of potential invaders have been regarded as the cheapest and most effective control strategies [4], where one particular interesting phenomenon may offer opportunities for early intervention: the occurrence of invasion-related lags [5,6]. An *invasion lag* is a prolonged period of time that is sometimes observed between the establishment of an alien species and the time point at which it becomes invasive, rapidly increasing in numbers and spreading geographically. This phenomenon has been documented for a large number of invasive plants [7,8], invertebrates [9], birds [10], fishes [11], amphibians [12] and reptiles [13], with invasion lag times lasting for years or even decades in some cases. To date, the underpinnings of invasion lags are little understood, and accordingly they are currently not predictable, rendering innocuous species and species that will become invasive indistinguishable [14].

Several theories have been proposed to explain the occurrence of invasion lags [15]. For instance, changes in the biotic or abiotic environment [5], in climate [16,17] or in human activity might at some point create conditions that are more favourable for invasion, allowing a seemingly benign established alien species to suddenly become invasive [18,19]. A perhaps more intriguing type of dynamics that can determine the length of an invasion lag, facilitating a switch from a low-frequency alien species with a limited spread to an invasive species with significant impact on the ecosystem, may stem from changes in the invasive population itself. One such possibility is via introduction of a new variant of an established species that is coincidentally better adapted to local conditions, or that provides the founder population with the genetic diversity necessary to overcome inbreeding depression [20–22]. However, we now have evidence that variation can also emerge within the founder population itself, which becomes more successful over time as it evolves in the new environment [23]. Thus, genetic or phenotypic adaptation may provide the necessary fitness advantage to the introduced species, increasing its invasion success [24]. This phenomenon has mostly been documented in plants [25–27], but it has also been observed in animals [28]; for example, cane toads in Australia have evolved increasingly longer legs, accelerating their invasive spread [29], and phenotypic plasticity has been found to contribute to invasion success in social insects [30,31].

Here, we propose an alternative explanation for the occurrence of invasion-related lags. Namely, we consider the possibility that adaptation in invasive species can be conferred by the acquisition of beneficial microbes. The importance of considering non-genetic (or ‘extra-genetics’) modes of adaptation has been highlighted in evolutionary biology [32–38], and the microbiome may provide an important route of adaptation [39,40]. With ‘microbially mediated adaptation’ we refer to the process by which a species’ accommodation of an environmental challenge is realized through the function of microbes that are found in or on it. Thus, for example, adapting to the presence of a previously unencountered pathogen might be via genetic adaptation of immune-related genes, butit could also occur via the action of a gut microbe that prevents the establishment of the pathogen. This is a case in which the host reaps adaptive value from a genetic change that is extra-genetic to the host—in the sense that the microbial function, by which the benefit is provided, is largely genetically encoded in the microbes’ genes—and the benefit is a product of the inter-species interaction [39,40].

It is increasingly recognized that host–microbiome interactions can shape host fitness and evolutionary potential, e.g. by increasing host tolerance to abiotic stress, by allowing the breakdown of local food sources or by protecting the host from pathogens [41–49]. Importantly, these responses can be extremely rapid. For example, a reduction in microbiome diversity in the gut of tadpoles can decrease host fitness and their tolerance to thermal stress within days [41], and the acquisition of a pesticide-degrading bacteria can confer immediate resistance to pesticides for bean bugs [42]. However, the current literature has focused on understanding how the presence or absence of certain microbes can affect host fitness, without explicitly considering how this fitness advantage may affect the host population dynamics. While some studies in plant-soil communities have explored the influence of microbiome-related dynamics on multi-species communities (e.g. [50–52]), studies in animal communities rarely identify the microbiome as a possible route of adaptation that is able to influence the host's ecological dynamics [43,53]. In particular, studies considering how variations in microbial communities may affect invasion have primarily dealt with the transmission of pathogens, rather than with the exchange of mutualistic microbes [54,55].

Microbial acquisition can occur through vertical transmission from parents to offspring or through different pathways of horizontal transmissions, such as direct contact between individuals, coprophagy (eating other individuals’ faeces), predation of younger individuals or pickup of microbes that survive an intermediate phase in the environment outside the host [56,57]. Horizontal transmission of microbes can occur not only between individuals of the same species but also between individuals of different sympatric species [58,59]. For example, predators are likely to be colonized by microbes from their prey [60,61], and social living can provide opportunities of microbial transmission between species [62–64].

Here, we suggest that a likely source of beneficial microbes for an introduced species is native hosts. We discuss the case in which invaders become better adapted to local conditions through the acquisition of mutualistic microbes from the microbiome of phylogenetically and ecologically similar co-occurring native species, and we present a theoretical framework that can be used to explore this hypothesis. Both factors are important: phylogenetic closeness increases the likelihood that the internal environment that the invasive provides to the microbes—in the gut, for example—would be appropriate for it; ecological similarities may be reflected in similar basic needs of the native and invasive species, and thus in native microbes having a similar adaptive potential for invasive species.

Beneficial microbes may, in principle, be acquired from the new environment that the invasive species reach. We suggest that this is unlikely, particularly with respect to microbiomes in internal body sites such as the gut, vagina, various glands and microbiome-specialized organs. This is because environmental microbes would rarely be able to prosper at these sites in which conditions are vastly different from the external environment, and that are in many cases evolutionarily adapted to creating selection pressures that would prevent such colonization (notably, this may not be true for externally exposed body sites such as skin and fur, where colonization by environmental microbes is more common, e.g. [57,65]). Even if environmental microbes do successfully establish, such facultative associations are likely to be of secondary importance for host fitness compared to co-evolved relationships between microbes and host.

Our study is part of a growing body of literature that aims to understand how social microbial transmission can shape the evolution and ecology of their hosts [40,47,58,59,66]. In our work, we choose to focus specifically on cases in which microbiome transfer has a positive impact on fitness, given that this scenario has received significantly less attention than the sharing of parasites or pathogens—in particular in the animal kingdom [67,68]. We acknowledge that if invasive hosts can acquire beneficial microbes from natives, we would expect that the acquisition of pathogens is also possible. The exchange of pathogens may also affect invasion dynamics by reducing competitiveness in natives or in invaders. One prominent such example is in the case of the invasive grey squirrel (*Sciurus carolinensi*), whose spread in Europe has been facilitated by the infection of the native population of red squirrels (*Sciurus vulgaris*) with squirrelpox: a highly pathogenic disease carried by grey squirrels, which appear to be immune to it [69]. Additionally, invasive hosts could also acquire microbes from native hosts. For a comprehensive listening of how different combinations of mutualistic and parasitic/pathogenic interactions between microbes and a newly introduced host may affect species’ competitive dynamics and invasion success and for a full theoretical treatment of these dynamics, we refer our reader to our recently developed framework [66] and, in general, to previous work in parasite ecology and plant invasion [50,70,71].

## 2. Model and methods

We formulate an ordinary differential equation model to study the coupled dynamics of a native population N competing with an introduced population I, whereby interactions are modelled according to the competitive Lotka–Volterra equations [72]. The populations experience logistic growth until reaching a certain carrying capacity, when competition between species can reduce or even reverse the growth (see electronic supplementary information, §1 for a complete mathematical analysis of the Lotka–Volterra equations).

We consider that some microbial species, found in the microbiome of native hosts, may be acquired by introduced individuals, and we explore the system’s dynamics under a range of parameters that govern this process. We consider that horizontal transmission can occur directly, through contact among individuals, or indirectly, with transmission mediated by the environment. This may include, for example, cases in which individuals of the invasive species utilize roosts or shelters that were previously occupied by native hosts, cases of coprophagy or situations where birds of the different species share sand or water bathing sites. We also posit that, once acquired, the microbes may be vertically and horizontally transferred within the introduced population. In our study, we do not differentiate either between microbes or between their locations within the host. Rather, with ‘microbiome’ we mean any collection of symbiotic microorganisms that increases fitness in its host. For simplicity, we treat the transmission of the microbiome as a single event that may or may not occur, although in reality we expect transmission of only few microbial species—but with potentially large effects on fitness.

We model this scenario of interest by splitting the introduced population I into the following two subgroups: the subpopulation that has not acquired microbes from native hosts ($I_{0}$) and the subpopulation that has acquired microbes from native hosts ($I_{m}$). Individuals can move from $I_{0}$ to $I_{m}$ by acquiring native microbes through interaction with natives (N) or through interaction with introduced individuals that have already acquired native microbes ($I_{m}$). Intraspecific competition between subpopulations $I_{0}$ and $I_{m}$ is also observed, where the total size of population I is limited by a fixed carrying capacity. Mathematically, we write:


(2.1a) $$\frac{dN}{dt}=\underset{logistic\;growth}{\underbrace{r_{n}N\left(1-\frac{N}{K_{n}}\right)}}-\underset{competition\;with\;I}{\underbrace{\alpha_{in}NI_{0}-\alpha_{mn}NI_{m}}}\;,$$

(2.1b) $$\begin{array}{l} \frac{dI_{0}}{dt}=\underset{logistic\;growth}{\underbrace{r_{i}I_{0}\left(1-\frac{I_{0}+I_{m}}{K_{i}}\right)}}-\underset{competition\;with\;N}{\underbrace{\alpha_{ni}I_{0}N}}-\underset{\begin{array}{c} microbial\;transfer\\ \text{(}N\to I_{0}\text{)} \end{array}}{\underbrace{\Lambda_{n}}}-\underset{\begin{array}{c} microbial\;transfer\\ \text{(}I_{m}\to I_{0}\text{)} \end{array}}{\underbrace{\Lambda_{m}}}, \end{array}$$

(2.1c) $$\frac{dI_{m}}{dt}=\underset{logistic\;growth}{\underbrace{r_{m}I_{m}\left(1-\frac{I_{m}+I_{0}}{K_{m}}\right)}}-\underset{competition\;with\;N}{\underbrace{\alpha_{nm}I_{m}N}}+\underset{microbial\;acquisition}{\underbrace{\Lambda_{n}+\Lambda_{m}}}\;,$$

The ability of a population to outcompete a population depends on its growth rate ($r_{j}$), on its carrying capacity ($K_{j}$) and on its competitive effect ($\alpha_{wj}$), where subscripts j and w stand for n, i or m. With ‘competitive ability’ we refer therefore to the set of traits of a population (in our model, the set of parameters $r_{j}$, $K_{j}$ and $\alpha_{wj}$) that characterize the growth of population j in the presence of population w, with populations j and w representing either the native or the introduced populations. A description of model variables and parameters is provided in table 1. The population that outcompetes the other is referred to as the ‘superior competitor’.

**Table 1.** Brief description of the variables and parameters of the system of equations in (equation 2.1a-c).

symbol	description
N	native population
$I_{0}$	introduced population (without native microbes)
$I_{m}$	introduced population (with native microbes)
$r_{j}$, with j=n,i,m	growth rate of population j
$K_{j}$, with j=n,i,m	carrying capacity of population j
$\alpha_{wj}$, with wj=ni,nm,in,mn	competitive effect of population w on population j
$\lambda_{j}$, with j=n,m	density-dependent microbiome transfer rate from population j

^a^ Subindex *n* refers to the native population N, subindex *i* refers to introduced population without native microbes (subpopulation $I_{0}$) and subindex *m* refers to the introduced population with native microbes (subpopulation $I_{m}$ ).

If the waiting time for a microbiome transfer event to happen is exponential, as commonly assumed in modelling, microbiome transfer can be simulated as a Poisson process with a rate that depends on the density-dependent microbiome transfer rate from natives to introduced individuals ($\lambda_{n}$) and on the size of the native and introduced populations (N(t) and $I_{0}(t)$, respectively). This implies that the number of introduced individuals that acquire native microbes in the time interval (t,t+dt) through inter-specific contact is a Poisson random variable $\Lambda_{n}(t)$, with rate $\gamma_{n}(t)=\lambda_{n}N(t)I_{0}(t)$, such that:


(2.2) $$\begin{array}{ccc} & \Lambda_{n}∼\mathrm{Pois}(\lambda_{n}N(t)I_{0}(t)dt)\;. & \end{array}$$

The density-dependent microbiome transfer rate $\lambda_{n}$ may depend on the factors that underlie the biology of transmission and host–microbe interactions. For instance, ecological similarity or phylogenetic relatedness between native and invasive hosts may increase the likelihood that native microbes may establish in an invasive host [73,74]. This may be owing, for example, to physiological similarity between the native and invasive hosts at the relevant body site in factors such as the structure and abiotic conditions in a host’s digestive tract. It may also be related to similarity in the availability of resources to the microbes, resulting from similar host diets or excretion of similar complex carbohydrates by the host's epithelial cells in the gut. Parameter $\lambda_{n}$ may also depend on the mode of transmission [56]: direct contact between hosts, e.g. through predation or coprophagy, may increase the likelihood of microbial acquisition by a new host [61,75], while indirect contact, e.g. through the use of the same sand or water for bathing or digging, may lead to a lower rate of microbial acquisition. Finally, $\lambda_{n}$ may depend on the microbes themselves, because not all microbes are equally likely to be transmitted or acquired [76]. Note that our model does not require microbiome transfer to happen fast. Here, we simply consider that microbiome transfer is possible and that in some instances it would provide adaptive value to the invasive host, as long as the rate $\lambda_{n}$ is larger than zero.

Once acquired, the microbiome can be transferred horizontally from $I_{m}$ to $I_{0}$ through the same modalities described above, at a rate that depends on the density-dependent microbiome transfer rate among introduced individuals ($\lambda_{m}$) and on the sizes of subpopulations $I_{0}$ and $I_{m}$. Again, as for $\lambda_{n}$, the value of parameter $\lambda_{m}$ should also depend on the mode of transmission and on the characteristics of the transferred microbes. The number of individuals that acquire native microbes through intraspecific contact can be described by a Poisson random variable $\Lambda_{m}(t)$, with rate $\gamma_{m}(t)=\lambda_{m}I(t)I_{0}(t)$, such that


(2.3) $$\Lambda_{m}∼(\lambda_{m}I_{m}(t)I_{0}(t)dt)\;.$$

We look at the situation in which a small number of introduced individuals are released into the environment while the native population is at its carrying capacity, and we discuss scenarios in which the introduced population is poorly adapted to local conditions before acquiring native microbes and better adapted afterwards. Below, we discuss how these scenarios are implemented in our model.

### (a) Microbial acquisition and adaptation

We consider that the acquisition of beneficial microbes can lead to an increase in the carrying capacity or in the growth rate of the introduced species (from $K_{i}$ to $K_{m}$, and from $r_{i}$ to $r_{m}$) or to changes in the competitive effect that native and invasive species have on one another (from $\alpha_{ni}$ and $\alpha_{in}$ to $\alpha_{nm}$ and $\alpha_{mn}$). An increase in the carrying capacity could occur if the acquired microbes lead to adaptations that confer a dietary or spatial niche expansion. Examples are microbes that aid the metabolic processing of local diet or microbes that lead to the breakdown of toxins and other secondary metabolites in food that allow for resource consumption [42,43,77]. Niche expansion through the mechanisms mentioned above can also affect competitive dynamics, broadening the overlap in the dietary and spatial niches of the invasive and the native species [77]. An increase in the population growth rate could occur, for example, if native microbes lead to resistance to local pathogens [46] or if acquiring microbes that lead to toxin or metabolite breakdown allows for increased longevity, which can happen, for example, if the accumulation of toxins has negative long-term effects that microbes can mitigate. Microbial associations can also confer protection from environmental perturbation, such as changes in temperature [41,45], which would also lead to an increase in the growth rate and eventually to an increase in carrying capacity if the protection conferred by the microbes allows the host to expand its niche.

The impact of changes in the carrying capacity, growth rate or competitive effect on the competitive dynamics of native and invasive species can be visualized by comparing the relative position of the nullclines in the phase planes of electronic supplementary material, figure S2 (see electronic supplementary information, §1). In some cases, changing any of these parameters would simply change the population sizes of the native or invasive species at equilibrium, or change the rate of a process of replacement that would have happened anyway; in others, it may make the difference between invasion success and invasion failure. For instance, increasing the growth rate of the introduced species can change a scenario in which invasive species are competitively excluded by natives into one of coexistence (see electronic supplementary material, figure S2). Specifically, we discuss three scenarios in which, prior to microbial acquisition from natives, invasive species are coexisting with natives (scenario A), competitively inferior to natives (scenario B) or competitively superior to natives (scenario C). In all scenarios, invasive species can coexist with (or competitively exclude) natives after microbiome acquisition has occurred. The mathematical analysis of scenarios A–C and the default parameters used for the simulations are presented in the electronic supplementary information, §§2 and 3. Simulations are run in MATLAB 2022b and the code is publicly available at https://figshare.com/articles/software/Code_microbiome_transfer/21977765.

#### (i) Scenario A: the timing of microbiome acquisition affects invasion lag times

We consider the case in which prior to microbiome transfer, the native and the introduced populations have reached an equilibrium of stable coexistence, whereby introduced individuals are few with respect to natives (i.e. $K_{n}<r_{i}/\alpha_{ni}$ and $K_{i}<r_{n}/\alpha_{in}$; see electronic supplementary material, figure S2). The acquisition of native microbes leads to either an increase in the carrying capacity of the introduced species or to an increase in its competitive effect on the native species, which results in the displacement (or size reduction) of the native population. If we consider that introduced and native species can coexist for a long time before a microbiome is transferred from one species to the other, analysis of this scenario provides insights into the possible role of microbial acquisition in driving a lag in biological invasion and into the impact of horizontal microbiome transfer between and within species on the invasion lag time.

#### (ii) Scenario B: the establishment of an introduced species is made possible by the transfer of microbes from native species

In this scenario, the introduced population cannot persist in the new environment and experiences a population decline after introduction, owing to being unable to attain positive population growth (modelled as considering $r_{i}<0$ and $dI_{0}/dt=r_{i}I_{0}$; see electronic supplementary information, §4), or owing to competition with well-adapted natives (i.e. $K_{n}>r_{i}/\alpha_{ni}$ and $K_{i}<r_{n}/\alpha_{in}$; see electronic supplementary material, figure S2). We consider that the acquisition of native microbes leads to an increase in the growth rate or a decrease in the competitive effect that natives have on the invasive species, rescuing the introduced species from extinction. These changes result in the displacement (or a reduction in size) of the native population. Analysis of this scenario provides insights into the probability that an introduced population will adapt and stably establish in a new environment, thanks to the transfer of microbes from natives.

#### (iii) Scenario C: the presence of natives allows the invasive population to thrive

We consider the case in which introduced individuals are superior competitors and natives are driven to extinction in the presence of an introduced population (i.e. $K_{i}>r_{n}/\alpha_{in}$ and $K_{n}<r_{i}/\alpha_{ni}$; see electronic supplementary material, figure S2). However, the introduced population also has a low carrying capacity (electronic supplementary material, figure S3; scenario C) owing to being poorly adapted to local conditions. An increase in the carrying capacity of the invasive species through the acquisition of native microbes can lead to an increase in the size of the invasive population at equilibrium. This means that if introduced individuals acquire the microbiome from natives before displacing them through competition, the introduced population will thrive; otherwise, their population size will remain small. Analysis of this scenario provides insights into the interplay of competitive ability, patch size and population densities in determining the circumstances under which microbiome-mediated adaptation is most likely to occur.

## 3. Results

### (a) Scenario A: the timing of microbiome acquisition affects invasion lag times

**Figure 1.**
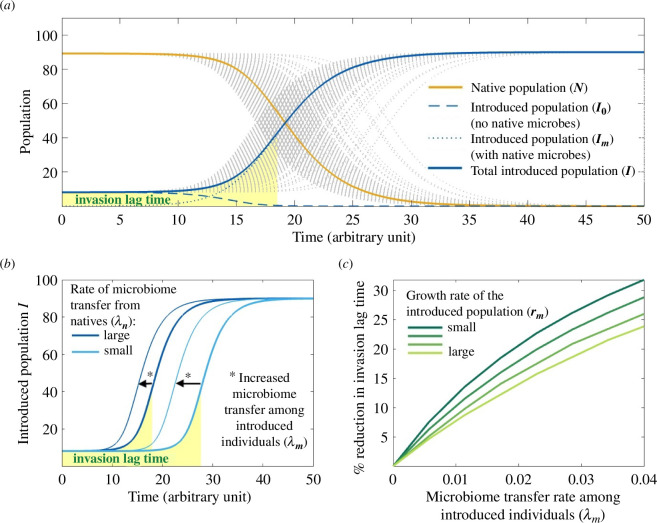
(*a*) Microbiome transfer from native to introduced individuals leads to adaptation of the introduced population (formulated in the model as the creation of a new subpopulation $I_{m}$, where $I_{m}+I_{0}$ represents the total introduced population I), which is competitively superior to the native population N. The invasion lag time is shaded in yellow. The orange and blue solid curves correspond to the mean average of 500 stochastic realizations of equation (2.1a–c) (grey dotted lines). (*b*) The invasion lag time increases when microbiome transfer events from natives to introduced individuals are rare (i.e. when $\lambda_{n}$ is small; light blue curves) and decreases when events are more frequent (i.e. when $\lambda_{n}$ is large; dark blue curves). (*c*) The occurrence of horizontal microbiome transfer among introduced individuals can decrease the invasion lag time, particularly when the growth rate of the introduced species $r_{m}$ is small.

We consider the situation in which stable coexistence of native and invasive species is observed, whereby the size of the introduced population is small owing to being poorly adapted to local conditions. Stable coexistence is maintained until microbes are transferred from a native to an invasive individual, and a new subpopulation $I_{m}$ is created that is better adapted than the introduced subpopulation $I_{0}$ (figure 1*a*). Eventually, the better-adapted subpopulation $I_{m}$ also displaces subpopulation $I_{0}$.

We call ‘invasion lag time’ the time interval occurring between species introduction and the inflexion point in the population growth of the introduced species, which depends on the time of the first microbiome transfer event. The lower the rate of microbiome transfer from natives to introduced individuals, the longer the invasion lag time, whereas horizontal microbiome transmission among introduced individuals can accelerate the spread of beneficial microbes within a population and decrease the invasion lag time (figure 1*b*). This effect is particularly prominent when the growth rate of the introduced population is low (figure 1*c*). In this case, it will take longer for the subpopulation with native microbes ($I_{m}$) to competitively displace the subpopulation without native microbes ($I_{0}$). Thus, subpopulation $I_{0}$ will still be largely represented in the total introduced population, slowing down the population growth of the introduced population as a whole. Horizontal microbiome transfer among introduced individuals can lead to a quicker spread of native microbes and to a faster conversion of $I_{0}$ into $I_{m}$.

### (b) Scenario B: the establishment of an introduced species is made possible by the transfer of microbes from native species

**Figure 2.**
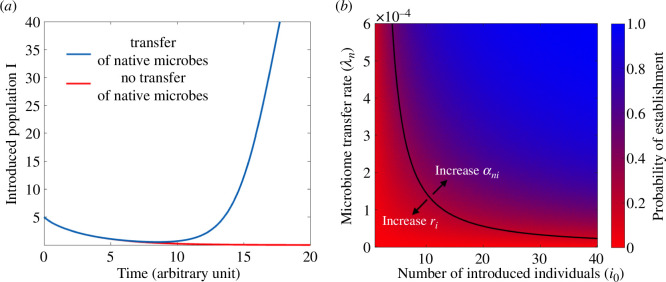
(*a*) A poorly adapted introduced population fails to establish if it does not timely acquire beneficial microbes from co-occurring natives (red curve). The transfer of beneficial microbes from natives to introduced individuals can confer adaptation to local conditions to the introduced population and rescue it from extinction (blue curve). (*b*) The probability of establishment of an introduced population (computed as the mean of 500 realizations) increases with increasing number of introduced individuals $i_{0}$ and with increasing density-dependent rate of microbiome transfer between the native and introduced population $\lambda_{n}$. The black curve represents the deterministic approximation derived in equation (3.1). Increasing the competitive effect of natives on introduced individuals $\alpha_{in}$ reduces their probability of establishment, while a larger growth rate of the introduced population $r_{i}$ increases it.

If the introduced population is poorly adapted to the local conditions, it may experience a decline in its population size after introduction owing to its own inability to sustain a positive population growth or owing to competition with better-adapted natives. Microbiome transfer from natives can facilitate the adaptation of the introduced species and ease its establishment. Thus, the persistence of an introduced population is made possible by the presence of co-occurring natives (figure 2*a*). Interestingly, the same native population that facilitates the establishment of an introduced species is subsequently likely to suffer from its spread. Indeed, if the now adapted introduced population experiences a rapid population growth, this may coincide with a reduction in the population size of natives and even with their displacement (electronic supplementary material, figure S3; scenario B). If microbial transfer occurs only after a long lag time, the resulting dynamics of invasion is similar to the lagged invasion discussed in scenario A (cf. figure 1*a* and electronic supplementary material, figure S5*a*).

Increasing the number of introduced individuals increases the probability that a timely microbiome transfer event will occur and facilitate the establishment of the introduced population (figure 2*b*). There are two reasons for this increase: (i) if the number of introduced individuals is large, it will take longer for the poorly adapted introduced population to die out This increases the probability that a microbiome transfer event will occur in time to confer adaptation to local conditions to the introduced population before its extinction. (ii) A large introduced population increases the rate of possible encounters between natives and introduced individuals, making a microbiome transfer event more likely to occur.

In the electronic supplementary information, §4, we derive a condition to determine under which circumstances microbiome exchange can facilitate the establishment of a poorly adapted introduced population. We obtain:


(3.1) $$\begin{array}{l} \lambda_{n}>\frac{\alpha_{ni}K_{n}-r_{i}}{K_{n}i_{0}\ln(i_{0})}\;,for\;K_{n}>r_{i}/\alpha_{ni}, \end{array}$$

where $\lambda_{n}$ represents an approximation for the minimal density-dependent microbiome transfer rate required, on average, for the establishment of the introduced population. Note that if the introduced population experiences a negative population growth even in the absence of competition with natives, equation (3.1) can be rewritten considering $\alpha_{ni}=0$ and $r_{i}<0$ (see electronic supplementary information, §4).

Equation (3.1) tells us that increasing the number of introduced individuals $i_{0}$ or the growth rate of the introduced population $r_{i}$ increases the probability that the introduced species will establish, while increasing the competitive effect of natives on introduced individuals $\alpha_{ni}$ will decrease it (figure 2*b*). Increasing the carrying capacity $K_{n}$ may increase or decrease the probability of establishment, depending on the strength of competitive interactions between natives and invaders and on the microbiome transfer rate. On the one hand, a large native population increases the probability that native microbes will be transferred to the introduced species in time to confer adaptation; on the other hand, a large highly competitive native population may cause the extinction of the introduced population before microbial acquisition (cf. electronic supplementary material, figures S6*b* and S7).

### (c) Scenario C: the presence of natives allows the invasive population to thrive

**Figure 3.**
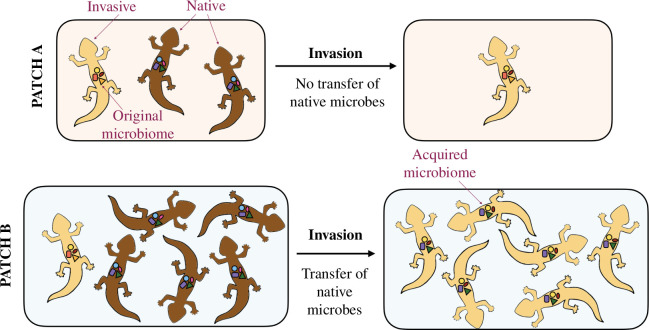
A native population (brown lizards) is competitively excluded by the introduction of a similar invasive species (yellow lizards). Patch A is smaller and presents a low density of natives, and the invaders displace the native population before the transfer of beneficial microbes from native to invaders can occur. In patch A, the invasive population remains, therefore, poorly adapted to the new environment and at low density. Patch B presents a larger patch size and a higher density of natives, which increase the likelihood that native microbes will be transferred to the invaders before natives are driven to extinction. The acquisition of native microbes confers local adaptation to the invaders, and their population in patch B grows larger than in patch A. The lizards here are chosen as an example to illustrate these dynamics. The mathematical analysis of this scenario is presented in the electronic supplementary information, §5.1.

Let us consider a certain patch in which a native population is present. Consider then that some highly competitive individuals of an invasive population are introduced to the patch. If introduced individuals have a higher competitive ability than natives, but are poorly adapted to local conditions, they may outcompete natives but remain present at low density after the invasion. If, however, invaders are conferred local adaptations through the acquisition of native microbes, their population may reach higher densities after the invasion. Thus, if the native population is displaced by the invaders before a microbiome transfer can occur, invaders will remain in low numbers; otherwise, their final population size will be larger (figure 3). Note that if the microbiome transfer rate is low, the increase in the size of the invasive population is expected to be observed only after a lag time (electronic supplementary material, figure S5*b*).

The probability of acquiring microbes from natives will depend on the population densities of natives and invaders within a patch, on the nature of their interactions and on the patch size. In the electronic supplementary information, §5, we show that microbiome-mediated adaptation may occur when the minimal average density-dependent microbiome transfer rate $\lambda_{n}$ satisfies


(3.2) $$\begin{array}{l} \lambda_{n}>\frac{2\sqrt{D_{i}(r_{i}-\alpha_{ni}K_{n})}}{\Delta x\;\tilde{n}\;\tilde{j}}\;for\;K_{n}<r_{i}/\alpha_{ni}. \end{array}$$

Equation (3.2) tells us that the transfer of native microbes is more likely to occur when the average densities of natives $\tilde{n}$ and invaders $\tilde{j}$ in a patch are large, when patch size Δx is large and when natives can slow down the growth of the invasive population through competition (i.e. $\alpha_{ni}$ is large enough, with $\alpha_{ni}<r_{i}/K_{n}$). Under these circumstances, it will take longer for the invaders to outcompete natives, increasing their chance of acquiring native microbes and becoming locally adapted.

High rates of dispersal of the invasive species may influence the dynamics in two opposing ways. On one hand, compared to a scenario in which dispersal is limited and the invasive species progresses in space as a moving front of invasion, increased dispersal creates more situations in which an invasive individual or an invasive population is surrounded by native hosts. This increases the rate of interaction between invasives and hosts and thereby the likelihood of microbiome transmission between them. However, if increased dispersal also has the effect replaceing of the native species more quickly, as discussed here (see figure 3), it might lead to faster displacement of natives, which overall reduces opportunities for microbiome exchange. In this case, within a patch, slow dispersal (i.e. low $D_{i}$) may offer more opportunities for microbiome exchange than fast dispersal because the interface between native and invasive populations is maintained for a longer time.

## 4. Discussion

It is increasingly recognized that rapid evolution driven by both genetic and non-genetic factors can alter invasion dynamics [29,34,35]. The microbiome has been proposed as a non-genetic mode of conferring adaptability to host species [39,40,53]; however, the consequences of this adaptation for community dynamics in the context of invasion remain largely unexplored. We suggest that the adaptation of an introduced population to local conditions can be mediated by the acquisition of beneficial microbes from similar co-occurring native species and that invasion can follow as a result.

Relatedness between invasive species and the recipient community is considered a weak predictor of invasion success [78–81]. On the one hand, similarities with natives may increase the likelihood that an invader’s traits will match the new environmental conditions, as suggested by the niche conservatism principle [82,83]. On the other hand, an invader may be more likely to suffer from direct competition with closely related natives in such a case, as stated by Darwin’s naturalization hypothesis [84–86]. Here we propose that, non-intuitively, invasion may be *facilitated* by the presence of co-occurring native species if the acquisition of beneficial pre-adapted microbes from the microbiome of natives can boost invaders’ fitness. Particularly, even when invaders are superior competitors, the acquisition of native microbes may confer local adaptations to an invasive population and facilitate its population growth and spread, including possible influence on the eventual steady-state abundances of the invasive and native species. The rate of encounter between natives and introduced individuals and thus the probability that an introduced species will acquire a beneficial microbiome should increase in the presence of larger native and founder populations (see figure 2). This also provides an additional explanation for the empirical observation that propagule pressure plays an important role in facilitating invasion [87]. Indeed, a large number of arriving propagules would increase the rate of native–invasive encounter and the respective rate of microbiome transmission during the crucial phase during which the invasive is struggling to establish.

The idea that invaders may benefit from established mutualistic associations between native hosts and their microbes has already been formulated in plant ecology, where the establishment of an introduced plant and its expansion in a new range can be facilitated by the presence of pre-existing mycorrhizal networks [50,88–90]. However, this concept is new to animal ecology, where inter-species horizontal transmission of mutualist microbes remains largely unexplored [56,67] and research that links microbiome acquisition and host adaptation in animals is promising [41,42,91], but still in its infancy.

In a rapidly changing world, in which connectivity is consistently increasing, opportunities for inter-specific microbial exchange to take place are plentiful. Exchange of microbes can be brought about, for example, through predation of native individuals or through coprophagy, a behaviour documented in many animals, including invasive lizards and rodents [92,93]. Alternatively, environmentally mediated exchange could occur at shared sand or water bathing sites, e.g. between the invasive Indian myna (*Acridotheres tristis*) and many native species in the mynas’ sunning, nesting and shelter sites, as well as their feeding grounds [94]. The extent to which such exchange may result in the successful establishment of the natives’ microbes in the invasive species and how these relationships translate to function are unknown, and further research is needed to discover whether inter-species sharing may provide significant adaptive value to the invasive species. It seems highly likely, however, that some microbial species that co-evolved over thousands of generations with a native host provide an adaptive value that may carry over to another host species that is related to it, such as a scenario in which a microbe that facilitates a certain carbohydrate’s breakdown in the gut of a native detritivore is picked up by a host species that belongs to the same ecological guild.

The number of studies comparing the microbiomes of native and invasive species in plants is growing [95,96], but only a few studies have focused on comparing the microbiomes of native and invasive animals [97,98]. A recent study [97] found that native mussels shared a substantial fraction of their microbiome with the co-occurring invasive species *Corbicula fluminea*, indicating that invasive mussels may host microbial communities that are obtained locally. Additionally, a few more studies have compared the microbiome of invaders in their native and invasive ranges [99,100] or in the population core and at the edges of their expansion range [101,102]. We suggest that such studies are necessary for understanding the possible importance of microbiome-mediated adaptation in general, as well as for testing the proposed hypothesis of adaptive microbiome pickup from native hosts as a mode of rapid adaptation. A complementary exploration of failed invasions may also be insightful: comparing between the microbiomes of an invasive species in cases where the species established and cases where its establishment ultimately failed [15] can highlight the microbiome’s potential role in this process.

In addition to influencing invasion dynamics, microbial acquisition may also contribute to facilitating species’ reintroduction into the wild [67,103,104]. Reintroduced populations share prominent characteristics with invasive ones: they are necessarily founded by individuals from a different environment—either from a population elsewhere in the wild or from a captive population, and they often go through demographic and genetic bottlenecks in the process. The latter often lead to low genetic diversity, making these populations particularly susceptible to demographic and environmental stochasticity [105,106] and more likely to suffer from inbreeding depression [105]. Phenotypic plasticity has been shown to help founder populations with low genetic diversity to maintain high fitness [107–109], and thus the extended phenotypic response that could be provided by the acquisition of native microbes may possibly help compensate for this lack in diversity and mediate the establishment success of small founder populations.

When microbial intake from other host species is beneficial, it would make sense to facilitate such acquisition prior to introduction. In Israel, for example, where several ungulates have been successfully reintroduced after having gone locally extinct [110,111], early exposure to fresh faeces of ungulates of the same ecological guild might have been beneficial to facilitate these reintroduction successes. This may in fact have occurred unintentionally, as the breeding cores of these species shared the facility with individuals of native species (ibex (*Capra nubiana*) and dorcas gazelle (*Gazella dorcas*)).

A concern that may justly be raised with respect to exposure of a reintroduced population to faeces of related speciesis that just as they may share beneficial microbes, they may share parasites and pathogens, and the exposure might put them at risk. Although this risk exists, we suggest that it would still usually be preferable to expose the reintroduced individuals to the microbiome of local hosts early on, while still in captivity. This is because such exposure will occur sooner or later, and be it harmful, beneficial or both, it would be advantageous to carry it out prior to release in the wild, in a controlled setting. However, the topic requires deep consideration, which is beyond the scope of the current study.

Our hypotheses could be tested experimentally in controlled conditions that emulate invasion scenarios, comparing the invaders’ fitness when faced with local conditions, with and without exposure to native species that may act as potential microbiome sources for local adaptation. Perhaps more interestingly, it may also be explored in invasive species that were introduced independently multiple times to sites that are disconnected. An example is the marbled crayfish (*Procambrus virginalis* [112]): this species has been introduced, in some cases, to multiple wetlands in the same region that are characterized by similar environmental conditions, but that differ in the native crustacean hosts that occur in them and that may function as native microbiome ‘donors’. Comparing the crayfishes’ fitness and invasive success among these sites and linking them to the microbiome site composition of the native and invasive crustaceans may thus be highly informative. We have recently detected and begun to study this situation in Israel, where marbled crayfish were found at several sites [113].

## 5. Conclusion

The need for developing theoretical frameworks to predict invasive potential when invaders evolve in their environment has been highlighted in several instances [14,24]; however, this call has largely remained unanswered. Here we present a mathematical model that sheds light on possible dynamics occurring if invaders evolve after their introduction, and we focus on the situation in which evolution is driven by the transfer of beneficial microbes from the microbiome of similar co-occurring native species. Our work presents a simple framework that sets the basis for broadening the conceptualization of microbiome-mediated dynamics and opens the door to further theoretical exploration and scientific discoveries.

## Data Availability

The paper has no data. The code used for the simulations is novel and publicly available at [114]. Supplementary material is available online [115].
